# Rapid trauma classification under data scarcity: an emergency on-scene decision model combining natural language processing and machine learning

**DOI:** 10.1007/s11517-025-03414-x

**Published:** 2025-07-11

**Authors:** Jun Tang, Tao Li, Liangming Liu, Dongdong Wu

**Affiliations:** 1 Department of Information, Daping Hospital, Army Medical University, Chongqing, 400042 China; 2 Department of Shock and Transfusion, State Key Laboratory of Trauma, Burns and Combined Injury, Daping Hospital, Army Medical University, Chongqing, 400042 China

**Keywords:** Trauma, Tiered medical treatment, Injury severity score, Natural language processing, Machine learning

## Abstract

Trauma has become a major cause of increased morbidity and mortality worldwide. In emergency response, the classification of injuries is crucial as it helps to quickly determine the criticality of the injured, allocate rescue resources rationally, and decide the priority order of treatment. However, emergency scenes are often chaotic environments, making it difficult for rescue personnel to collect complete and accurate information about the injured in a short period. The combination of artificial intelligence and emergency rescue is gradually changing the rescue model, improving the efficiency of rescue operations. We selected data from 26,810 trauma patients admitted to Chongqing Daping Hospital between 2013 and 2024. We propose a fast tiered medical treatment method with a two-layer structure under emergency limited data conditions, which integrates natural language processing (NLP) and machine learning (ML) techniques. The tiered medical treatment model utilizes NLP to capture semantic features of unstructured text data, while utilizing four ML algorithms to process structured numerical data. Additionally, we conducted external validation using 245 data entries from the Chongqing Emergency Center. The experimental results show that gradient boosting and logistic regression have the best performance in the two-layer ML algorithms. Based on these two algorithms, our model outperformed the multilayer perceptron (MLP) model on the test dataset, achieving an accuracy of 91.17%, which is 4.33% higher than that of the MLP model. The specificity, F1-score, and AUC of our model were 97.06%, 86.85%, and 0.949, respectively. For the external dataset, the model achieved accuracy, specificity, F1-score, and AUC of 87.35%, 95.78%, 80.37%, and 0.848, respectively. These results demonstrate the model’s high generalizability and prediction accuracy. A model integrating NLP and ML techniques enables rapid tiered medical treatment based on limited data from the emergency scene, with significant advantages in terms of prediction accuracy.

**Graphical Abstract:**

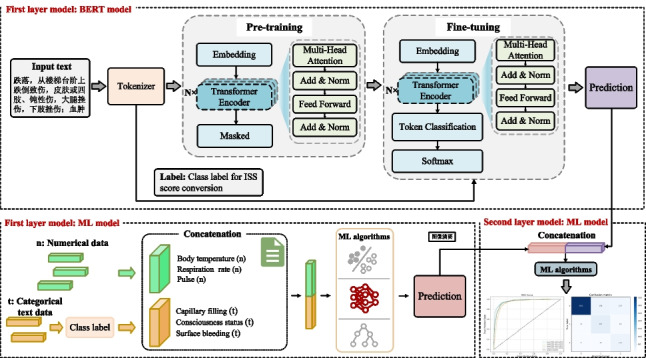

## Introduction

With the increasing frequency of traffic accidents and the exacerbation of natural disasters, injuries have become a leading cause of morbidity and mortality worldwide. According to the World Health Organization’s 2022 report, approximately 4.4 million people die each year as a result of such events, while tens of millions more suffer non-fatal injuries. In disaster relief, injury classification plays a vital role in improving the efficiency and effectiveness of treatment. Injury classification is a comprehensive assessment based on several indicators, such as the physiological state of the injured person, the region of the injury, the amount of blood loss, and vital signs. This classification helps rescuers quickly judge the severity of injuries, allocate resources reasonably, and determine the priority treatment order.

The abbreviated injury scale (AIS) is an authoritative standard in trauma assessment. AIS is based on anatomical injuries and uses a precise coding and grading system to effectively assess the severity of patient injuries, providing a solid basis for clinical decision-making [1]. AIS forms the foundation of the injury severity score (ISS) system, which is widely used in professional rescue and medical fields to quantify the severity of injuries [2]. The ISS system evaluates injury severity by scoring the degree of damage to different body regions, such as the head, chest, and abdomen. The ISS scoring range is typically 1–75 points, with ISS < 16 defined as mild injuries; ISS ≥ 16 and < 25 as moderate injuries, with a possible mortality rate of less than 10%; ISS ≥ 25 and < 50 as severe injuries with a significant risk of death; and ISS ≥ 50 as critical injuries, with a low probability of survival and nearly imminent death [1]. Therefore, the ISS score is widely used for mortality prediction [3–5]. Currently, the initial examination and classification of patients are primarily conducted by medical personnel according to relevant regulations and ISS scoring standards. This method requires a large number of medical personnel with corresponding professional medical knowledge.

The integration of artificial intelligence (AI) and emergency rescue [6–9] is fundamentally changing the traditional rescue model, making it more efficient and safe. AI can process large amounts of information from disaster sites, providing faster and more accurate intelligent decision support for rescue personnel. This helps them develop reasonable rescue plans and action strategies, thereby reducing losses caused by disasters. In terms of patient classification [10, 11], AI can automatically prioritize patients by analyzing their physiological indicators and injury descriptions. For example, Li et al. [12] built a hierarchical intelligent emergency treatment system based on 5G and AI technology, expanding hospital service capacity and resources outside the hospital through the Internet and intelligent means. This addresses current challenges in emergency medicine, such as difficulties in data collection and insufficient intelligence in auxiliary diagnosis.

Despite the great potential of AI in the field of casualty classification, existing models still face challenges when applied in disaster scenes. One major issue is the difficulty in obtaining input data for AI models. Disaster sites often have harsh environments and chaotic information, making it difficult for rescue personnel to collect complete and accurate information about the injured in a short period. This limits the application of existing AI models in the field and prevents them from fully leveraging their advantages.

Therefore, to address the limitations of existing models, we propose a fast tiered medical treatment method with a two-layer structure under emergency limited data conditions. Specifically, our model integrates natural language processing (NLP) and machine learning (ML) techniques. It aims to capture deep semantic features of unstructured text data through the NLP model by integrating information from different data sources while utilizing ML algorithms to process structured numerical data to improve model prediction accuracy. Additionally, we adopted a hierarchical fusion strategy, where each layer is responsible for specific data processing and prediction tasks.

## Methods

### Ethics approval

The data used in this study were reviewed and approved by the internal review board of Chongqing Daping Hospital, with informed consent from patients waived. Ethics committee approval number: 2024_219.

### Patients and dataset

**Fig. 1:**
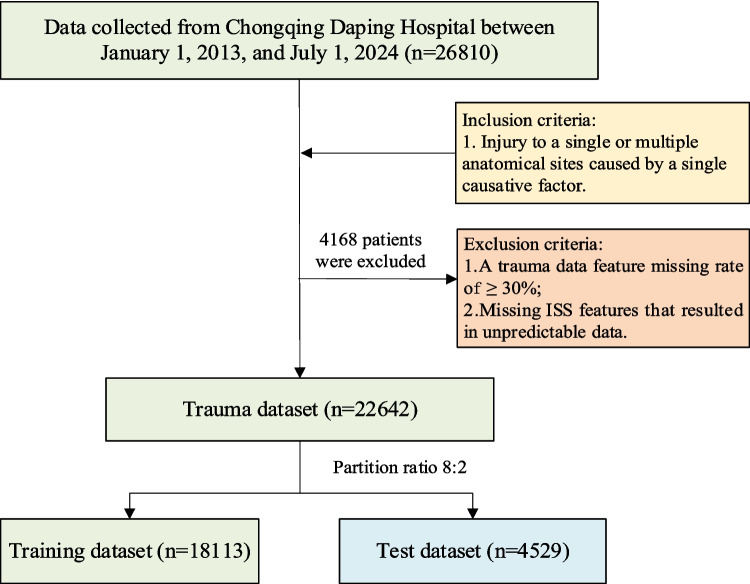
Patient selection process diagram

We retrospectively collected trauma data from 26,810 patients at Chongqing Daping Hospital between January 1, 2013, and July 1, 2024. The inclusion criteria were injuries to single or multiple anatomical sites caused by a single causative factor. The exclusion criteria included a trauma data feature missing rate of ≥ 30% and missing ISS features that resulted in unpredictable data. Based on these criteria, 22,642 entries met the criteria. Figure 1 illustrates the process of patient inclusion and exclusion.

The trauma data used in this study included patient injury descriptions, present illness history, injury region, injury types, body temperature, respiration rate, pulse, systolic blood pressure, diastolic blood pressure, respiratory status, capillary filling, eye-opening response, speech response, consciousness status, chest and abdominal tenderness, chest penetrating injury, surface bleeding, and ISS score features. Among these, the ISS score was defined as the outcome label of the model. We categorized injuries as follows: ISS score < 16 as mild injury, 16 ≤ ISS < 25 as moderate injury, and ISS ≥ 25 as severe injury, resulting in three categories. The remaining 17 features are used as input features for the model, selected based on their clinical relevance and feasibility for rapid acquisition in disaster rescue scenarios. These features can be quickly obtained through patient interviews or simple on-scene measurements. Throughout the data pre-processing and subsequent research stages, the number of features remains unchanged.

We divided the dataset (*n* = 22,642) into a training dataset and a testing dataset based on stratified sampling technique in an 8:2 ratio, with 18,113 entries in the training dataset and 4529 entries in the test dataset. Additionally, we used an external dataset from the Chongqing Emergency Center, consisting of 245 entries that met the inclusion and exclusion criteria. Table 1 provides a detailed list of demographic characteristics and injury information for these datasets.

**Table 1:** Demographic and injury characteristics of patients in the datasets

Variables	Training dataset	Test dataset	External dataset
Total number of patients	18,113	4529	245
Age ranges (IQRs)	1–102 (33)	1–98 (33)	14–97 (31)
Male (%)	11,616 (64.1)	2929 (64.7)	157 (64.1)
Mechanism of injury (%)			
Falls	9772 (54.0)	2579 (57.0)	96 (39.2)
Traffic accidents	3264 (18.0)	702 (15.5)	112 (45.7)
Sports injury	583 (3.2)	64 (1.4)	5 (2.0)
Sharp weapon injury	298 (1.6)	76 (1.6)	22 (9.0)
Other	4196 (23.2)	1108 (24.5)	10 (4.1)
Severity of injury (%)			
Mild injury	15,800 (87.2)	3896 (86.0)	228 (93.1)
Moderate injury	1384 (7.7)	399 (8.8)	14 (5.7)
Severe injury	929 (5.1)	234(5.2)	3 (1.2)

In the training, test, and external datasets, the interquartile ranges (IQRs) of age were 33 years, 33 years, and 31 years, respectively. This indicates that the age distribution is relatively concentrated and shows little variation across different datasets. There is also no significant difference in gender distribution.

Falls account for the greatest proportion of the training and test datasets (approximately 55%), whereas traffic accidents account for the greatest proportion of the external dataset (45.7%). This difference reflects the differences in environmental and population characteristics between the datasets. Among the three datasets, patients with mild injuries accounted for the largest proportion (87.2% in the training dataset, 86.0% in the test dataset, and 93.1% in the external dataset).

### Construction of a tiered medical treatment model

**Fig. 2:**
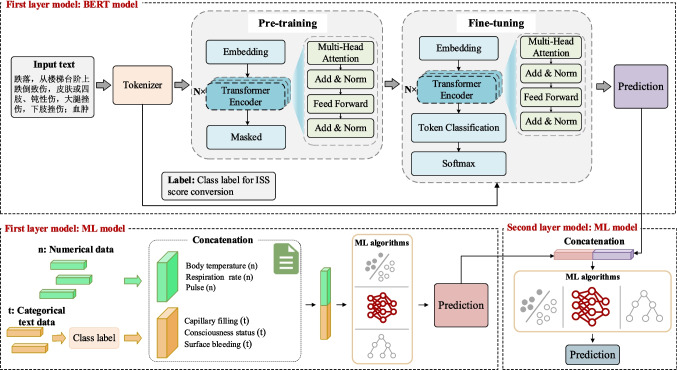
Tiered medical treatment model architecture

As shown in Fig. 2, our tiered medical treatment prediction model with a two-layer architecture integrates NLP and ML techniques. In the first layer, the model aims to integrate information from different data sources. On the one hand, it utilizes the BERT [13] model to capture semantic features from unstructured text data. On the other hand, ML algorithms are used to process structured numerical data. In the second layer, the model concatenates the output probabilities from the two sub-models in the first layer and inputs the concatenated results into another ML model to obtain the final ISS severity grading prediction.

(1) First layer model: BERT model.

For text data, we employ a BERT model based on NLP and fine-tune it to better suit our dataset and prediction tasks. This model implements dynamic adjustment of masking strategies and enhanced text encoding to learn contextual information from trauma patient data. This approach allows the model to capture both forward and backward contextual information in the input sequence, thereby gaining a deeper understanding of the text data.

Specifically, the vocabulary used for training includes the patient’s injury descriptions, present illness history, injury region, and injury type information, along with necessary special markers. The entire text sequence is treated as a sentence, identified by start and end markers, and padded to a uniform length. During pre-training, the BERT model receives input sequences and extracts contextual feature representations. These features are then passed to a multivariate classification head for fine-tuning, generating prediction probabilities for each ISS score category. We use the cross-entropy loss function to calculate the difference between the predicted probability distribution and the true ISS score label. Through the BERT model, we transform unstructured text data into high-dimensional, semantically rich vector representations, which serve as input features for subsequent ML models.

(2) First layer model: ML model.

We first convert categorical text data (e.g., respiratory status, capillary filling, eye-opening response, speech response, consciousness status, chest and abdominal tenderness, chest penetrating injury, and surface bleeding) into a format suitable for ML model processing, These data are concatenated with the original numerical data (e.g., body temperature, respiration rate, pulse, systolic blood pressure, diastolic blood pressure). We employed four ML algorithms: logistic regression, support vector machine, gradient boosting, and random forest algorithm [14]. These algorithms were chosen because they perform well in classification tasks and are relatively robust, with less sensitivity to hyperparameter selection compared to some other commonly used models. Each algorithm has its strengths and can provide stable predictive performance across different datasets and feature spaces.

The concatenated data were divided into training and validation datasets in an 8:2 ratio to obtain the ISS score category prediction probabilities. During algorithm implementation, we optimized the parameters of each algorithm to ensure optimal performance on our dataset.

(3) Second layer model: ML model.

In the second layer, we concatenate the output probabilities from the two sub-models in the first layer and input them into the ML algorithm for the final ISS classification prediction. The output probability of the first layer reflects the predictive contribution of both unstructured text data and structured numerical data to ISS severity grading. By concatenating these probabilities, we obtain a more comprehensive feature representation that enhances the accuracy of the final prediction.

## Results

### Model training and implementation details

The BERT model was trained via early stopping, with training concluding after the validation loss did not improve after ten epochs. The tiered medical treatment model was trained on a computing cluster with 48 GB of memory and one GPU (NVIDIA RTX A6000). We implemented the model via Python (version 3.8.19), PyTorch (version 2.3.1), NumPy (version 1.24.4), Pandas (version 2.0.3), and Scikit-learn (version 1.3.2).

### Overall predictive performance of the tiered medical treatment model

We used AUC, accuracy, specificity, and F1-score as evaluation metrics. For the AUC metric, we employed the macro-average method. To validate the performance of our tiered medical treatment model, we compared it with a multilayer perceptron (MLP) model [15], which concatenates different types of data features as input and directly outputs the final ISS prediction results. Table 2 shows the average and standard deviation (SD) results of five randomly repeated experiments on the test dataset.

**Table 2:** Comparison of model prediction performance. SD, standard deviation

Model	AUC (SD)	Accuracy (SD)	Specificity (SD)	F1-score (SD)
MLP	0.500 (< 0.01)	86.84 (0.06)	95.61 (0.03)	60.99 (0.06)
First layer model: BERT model	0.901 (0.01)	90.97 (0.15)	96.89 (0.12)	82.60 (0.11)
First layer model: ML model	0.877 (< 0.01)	89..25 (0.10)	96.42 (0.07)	77.80 (0.07)
Second layer model: ML model	0.949 (< 0.01)	91.17 (0.08)	97.06 (0.06)	86.85 (0.08)

In the first layer of our model, we tested four ML algorithms for the ML model component: logistic regression, support vector machine, gradient boosting, and random forest. The results indicated that the gradient boosting algorithm performed the best. Gradient boosting, an ensemble learning method, typically achieves superior performance when dealing with unbalanced data. Therefore, the results shown in Table 2 correspond to the gradient boosting model. These results were also used as input for the second layer model. For the second layer model, we applied a logistic regression algorithm, which achieved the best performance.

As seen in Table 2, our tiered medical treatment model achieved the best performance across all metrics. Specifically, its accuracy, AUC value, specificity, and F1-score reached 91.17%, 0.949, 97.06%, and 86.85%, respectively. The accuracy of the MLP model is 86.84%. This represents a 4.33% improvement in accuracy, demonstrating the effectiveness of our layered fusion strategy in enhancing ISS score prediction accuracy. Notably, both sub-models in our first layer (BERT and ML) outperform the MLP model individually. In addition, the SD indicates that our model has relatively small fluctuations.

**Fig. 3:**
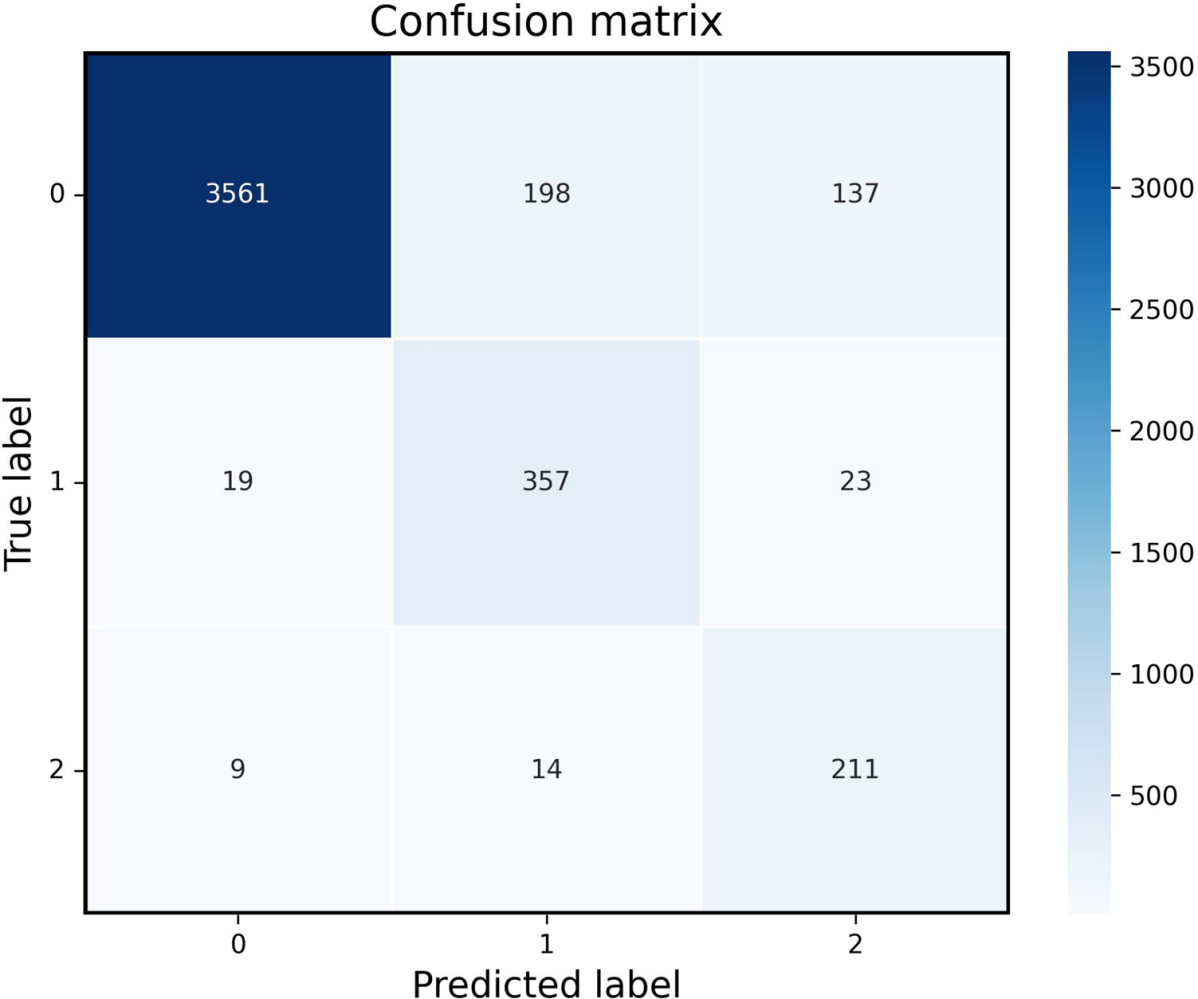
Visualization of confusion matrix in the tied medical treatment model

The performance of the tiered medical treatment model on the test dataset was evaluated in detail using a confusion matrix, and the results are shown in Fig. 3. From the confusion matrix, it can be seen that the model performs differently in different categories: Mild injury category (0): the model correctly predicted 3561 samples, incorrectly predicted 198 samples as moderate injury, and incorrectly predicted 137 samples as severe injury. Moderate injury category (1): The model correctly predicted 357 samples, incorrectly predicted 19 samples as mild injury, and incorrectly predicted 23 samples as severe injury. Severe injury category (2): The model correctly predicted 211 samples, incorrectly predicted 9 samples as mild injury, and incorrectly predicted 14 samples as moderate injury. Overall, the tiered medical treatment model achieved the highest accuracy in predicting mild injury and performed well in classifying the severity of injuries.

In the first layer, the BERT model slightly outperformed the ML model. This may be attributed to the BERT model’s superior ability to process unstructured textual data and capture information relevant to trauma severity. However, the two sub-models likely captured different types of information. When integrated into the layered model, the second layer was able to combine this distinct insight more effectively, thereby improving overall performance.

**Fig. 4:**
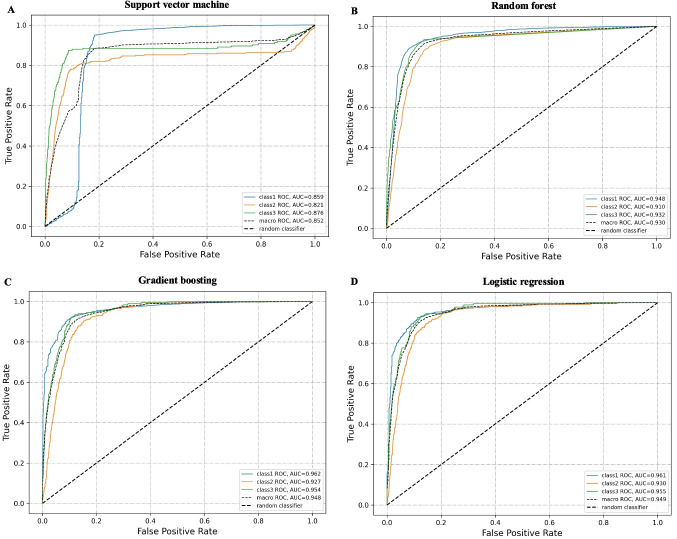
ROC curves on the test dataset

The receiver operating characteristic (ROC) curves for the second layer ML model on the test dataset are shown in Fig. 4. We visualized the ROC curves for all categories as well as their macro average ROC curve. By comparing the AUC values of different categories, we can assess whether there are significant differences in the model’s performance across these categories. The macro-average ROC curve is obtained by averaging the ROC curves of all categories, providing a comprehensive overview of the model’s overall performance in multi-class classification tasks.

Specifically, among the four algorithms tested, the logistic regression algorithm achieved the best results. The AUC values for the three categories were 0.961, 0.930, and 0.955, respectively, while the macro-average AUC value was 0.949. These high AUC values, which are close to 1, further demonstrate the model’s strong ability to distinguish between positive and negative samples, maintaining excellent performance across almost all possible classification thresholds.

Despite the presence of data imbalance in our test dataset, the ROC curves and AUC values for the three categories were relatively close to each other. This indicates that our model’s performance is relatively stable across different categories without significant bias.

### External validation

We conducted external validation of the constructed BERT model using data from 245 trauma patients at the Chongqing Emergency Center. This external dataset has a data structure similar to that of the training dataset. The experimental results for the external dataset are shown in Table 3, with accuracy, AUC value, specificity, and F1-score of 87.35%, 0.848, 95.78%, and 80.37%, respectively. Compared with the test dataset, the performance of the BERT model slightly decreased on the external dataset. This decline may be related to differences in data distribution between different medical institutions. However, the overall performance remains satisfactory, indicating that the BERT model has strong generalization ability.

**Table 3:** Performance of the BERT model on the test and external datasets

Dataset	AUC	Accuracy	Specificity	F1-score
Test dataset	0.949	91.17	97.06	86.85
External dataset	0.848	87.35	95.78	80.37

## Discussion

### Principal findings

In this study, we address the complexity and urgency of wounded classification in disaster relief and propose a method to achieve rapid-tiered medical treatment based on limited data available at the emergency scene. By integrating NLP and ML techniques, our model aims to improve the accuracy and efficiency of casualty classification in chaotic and complex disaster environments. Experimental results demonstrate that our tiered medical treatment model outperforms the traditional MLP model in terms of accuracy, specificity, F1-score, and AUC value, thereby validating the effectiveness of the hierarchical fusion strategy.

The excellent performance of our model can be attributed to its ability to handle diverse data types effectively. Data collected at disaster scenes are often a mixture of unstructured and structured information, including physiological indicators of the injured and descriptions of their injuries. By leveraging the NLP model (e.g., BERT) to process unstructured text data, we can capture implicit information relevant to the severity of trauma that may be overlooked by traditional methods. Meanwhile, structured numerical data are processed using advanced ML algorithms (e.g., gradient boosting), which further enhance the model’s predictive accuracy. This multimodal data processing approach enables our model to gain a more comprehensive understanding of the injured individuals, thereby facilitating more accurate classification decisions. Moreover, the hierarchical fusion strategy employed in our model plays a crucial role in its overall performance. By decomposing the data processing and prediction tasks into multiple layers, with each layer focusing on a specific aspect, the model achieves improved accuracy and robustness. In the first layer, the BERT model processes unstructured textual data, while the ML model handles structured numerical data, generating preliminary prediction results. These results are then fed into the second layer, where they are integrated and refined to produce more accurate ISS score predictions. This layered structure not only enhances the model’s prediction accuracy but also makes it more flexible and stable when dealing with complex and dynamic data environments.

The experimental results show that for the four ML algorithms, the gradient boosting algorithm performs the best in the first layer model, while the logistic regression algorithm performs the best in the second layer model. The gradient boosting algorithm is a powerful ensemble learning method that gradually optimizes prediction results by constructing a series of decision tree models. In the first layer, the algorithm is mainly used to process structured numerical data, such as physiological indicators of injured individuals, and capture nonlinear relationships and complex patterns within them. It builds decision trees iteratively, with each new tree dedicated to correcting the prediction errors of the previous tree, gradually improving the accuracy of the model. The logistic regression algorithm is a simple and effective classification algorithm, particularly suitable for processing preliminary processed data and making final classification decisions. In the second layer, the algorithm is mainly used to integrate and refine the prediction results generated in the first layer, including the unstructured text data processed by the BERT model in the first layer and the structured numerical data processed by the gradient boosting algorithm. It generates the final classification decision by linearly combining these features and applying logical functions to map the results to a probability space.

The significant advantage of this study is the large sample size and reliable test data, with the dataset containing information from a large sample cohort of trauma patients. This extensive and diverse dataset provides robust training material for deep learning applications, which is crucial to ensuring model performance.

In this study, we chose to use the hold-out method to validate the model at an 8:2 ratio instead of k-fold cross-validation, which was based on multiple considerations. Firstly, the dataset of this study has a large sample size, and using the hold-out method can ensure that the model is fully trained on rich data and learns complex patterns and patterns in the data. Meanwhile, a larger test set can also provide more representative and stable results for model performance evaluation, which helps to prepare and grasp the generalization ability of the model. Secondly, although k-fold cross-validation can effectively utilize limited data resources in small dataset scenarios, its advantages are not significant in large dataset scenarios. On the contrary, k-fold cross-validation significantly increases computational and time costs. In this study, considering the size of the data and the requirements for computational efficiency, the hold-out method can ensure the effectiveness of model evaluation while completing the experimental process more efficiently. Again, to address the issue of data imbalance, we employed stratified sampling techniques to ensure that the sample proportions of each category in the training and testing sets were consistent with the overall dataset, thereby mitigating the impact of data imbalance on model evaluation. In addition, we conducted five random repeated experiments and took the average as the evaluation result, further improving the stability and reliability of the evaluation and reducing evaluation errors caused by randomness.

The model’s performance on the test dataset was superior to that on the external dataset. This discrepancy may be related to distributional differences between the datasets that can affect the model’s generalizability. The external dataset had a significant proportion of missing features, such as capillary filling and chest penetrating injury. This lack of information likely degraded the model’s performance. Moreover, ISS scores often rely on the subjective judgment and extensive clinical experience of medical professionals. The external data originated from the Chongqing Emergency Center, which may have different data collection methods, injury patterns, and coding standards compared to the original data from Daping Hospital. These differences likely contributed to the increased difficulty in using the model for accurate predictions. Therefore, inconsistencies between datasets and missing features are likely the main reasons for the model’s decreased performance on the external dataset.

AI models are increasingly being employed in medicine, with many leveraging ISS scores to predict morbidity, mortality, and prognostic outcomes. However, the ISS scoring system is a highly refined and complex grading framework that encompasses injuries across all body regions and scores them based on the severity of the injury. Our tiered medical treatment model may serve as a powerful tool for injury severity classification studies. While the independent calculation of ISS scores and comprehensive medical data by trained healthcare professionals remains the gold standard in the field, obtaining ISS scores can become impractical in certain situations, such as when the number of patients is large or when detailed medical records are difficult to obtain. This is particularly true during the pre-hospital emergency and transport phases, where rapid and accurate injury assessment is especially challenging due to limited resources and environmental constraints. In such cases, our tiered medical treatment model can provide highly accurate predictions of ISS severity for individual patients.

Our model has a wide range of potential applications. It is not only applicable to large general hospitals but also to all types of medical institutions and emergency centers, particularly in remote areas with limited resources or in emergency rescue situations. The model can rapidly provide injury assessments and offer robust support for medical decision-making. Moreover, with the continuous advancement of medical informatization and data collection technologies, the model can be further integrated into existing medical information systems to achieve more intelligent and personalized medical services.

Our research differs from previous studies in several key aspects. Our dataset was collected from Chongqing Daping Hospital and Chongqing Emergency Center, containing 17 features specifically selected for their clinical relevance and rapid accessibility in disaster rescue scenarios. This makes our dataset unique and not directly comparable to datasets used in other studies. Our research methodology and task are also significantly different from previous work. We propose a novel two-layer model architecture that combines NLP models (BERT) with machine learning algorithms such as gradient boosting and logistic regression. This method aims to effectively handle the multimodal characteristics of our dataset and improve the accuracy and sensitivity of trauma severity classification. Given the differences in dataset, task, and methods, we believe that a direct comparison with previous research may not be appropriate. However, we have listed in Table 4 a comparison of our study with other related studies in terms of task, sample size, evaluation metrics, and methods. This table highlights the unique aspects of our research and provides context for why direct comparisons are challenging. We believe that our approach offers valuable insights and advancements in the field of trauma severity classification, particularly in disaster rescue scenarios.

**Table 4:** Comparison with previous studies in terms of sample size, evaluation metrics, and methods

Study	Task	Sample size	Evaluation metrics	Methods
Study A [5]	Prediction of trauma mortality	1052	Resource utilization, mortality	Retrospective chart, database review
Study B[11]	Triage, classification model	Not applicable (Literature review)	AUC, sensitivity, specificity, accuracy	ML, NLP
Study C[12]	Classification of splenic trauma	2225	AUC, sensitivity, specificity, negative and positive predictive value	Deep learning
Our study	Trauma classification	26,810 + 245	AUC, accuracy, specificity, F1-score	NLP and ML fusion

### Limitations and future works

Despite achieving good results, our tiered medical treatment model still has room for improvement. For example, one could explore the use of more advanced pre-trained large language models (e.g., GPT series [16]) instead of BERT, or investigate alternative numerical data processing methods to further enhance model performance. Additionally, data imbalance impacts model performance. In our dataset, there were significant disparities in the proportions of injuries across different severity levels, which may have contributed to the model’s weaker performance in predicting a small number of categories (e.g., moderate and severe injuries). In the future, we plan to collect more data and explore more methods to address imbalanced data, such as data augmentation, stratified hold-out, and resampling techniques, to improve the model’s generalization ability. Lastly, the complexity and uncertainty of disaster scenes place higher demands on the real-time performance and robustness of the model. In practical applications, the model needs to classify a large number of injured individuals quickly and accurately predict their ISS scores. Therefore, we need to further optimize the model’s computational efficiency and stability to ensure it operates reliably in complex and dynamic disaster environments.

## Conclusions

Our proposed tiered medical treatment model can achieve injury assessment and tiered medical treatment based on limited data from the emergency scene. By integrating NLP and ML technologies, the model achieved an accuracy of 91.17% in predicting the severity of injury. As a deep learning and data-driven prediction model, it exhibits a high degree of objectivity and consistency, effectively reducing human bias and workload, while improving the reliability and accuracy of evaluation results.

In the future, we plan to continuously optimize the model to adapt to a wider range of medical scenarios and data types. Specifically, future research will explore the use of larger language models that are more advanced than BERT to enhance text understanding capabilities and achieve optimal performance in complex dynamic environments at disaster scene. At the same time, we will also strive to solve the problem of data imbalance through data augmentation and resampling techniques. These works will aim to enhance the robustness and real-world applicability of the model.
